# Cancer Immunotherapy: Silencing Intracellular Negative Immune Regulators of Dendritic Cells

**DOI:** 10.3390/cancers11010108

**Published:** 2019-01-17

**Authors:** Yao-Hua Liu, I-Jeng Yeh, Ming-Derg Lai, Kuan-Ting Liu, Po-Lin Kuo, Meng-Chi Yen

**Affiliations:** 1 Department of Emergency Medicine, Kaohsiung Medical University Hospital, Kaohsiung Medical University, Kaohsiung 807, Taiwan; 980542@ms.kmuh.org.tw (Y.-H.L.); 910201@ms.kmuh.org.tw (I.-J.Y.); 890077@ms.kmuh.org.tw (K.-T.L.); 2 Department of Biochemistry and Molecular Biology, College of Medicine, National Cheng Kung University, Tainan 701, Taiwan; a1211207@mail.ncku.edu.tw; 3 Institute of Basic Medical Sciences, College of Medicine, National Cheng Kung University, Tainan 701, Taiwan; 4 School of Medicine, College of Medicine, Kaohsiung Medical University, Kaohsiung 807, Taiwan; 5 Graduate Institute of Clinical Medicine, College of Medicine, Kaohsiung Medical University, Kaohsiung 807, Taiwan; kuopolin@seed.net.tw

**Keywords:** dendritic cells (DCs), short-hairpin RNA (shRNA), small-interfering RNA (siRNA), intracellular negative immune regulator, cancer

## Abstract

Dendritic cells (DCs) are capable of activating adaptive immune responses, or inducing immune suppression or tolerance. In the tumor microenvironment, the function of DCs is polarized into immune suppression that attenuates the effect of T cells, promoting differentiation of regulatory T cells and supporting tumor progression. Therefore, blocking negative immune regulators in DCs is considered a strategy of cancer immunotherapy. Antibodies can target molecules on the cell surface, but not intracellular molecules of DCs. The delivery of short-hairpin RNAs (shRNA) and small-interfering RNAs (siRNA) should be a strategy to silence specific intracellular targets in DCs. This review provides an overview of the known negative immune regulators of DCs. Moreover, a combination of shRNA/siRNA and DC vaccines, DNA vaccines in animal models, and clinical trials are also discussed.

## 1. Introduction

Dendritic cells (DCs) are the most potent type of antigen-presenting cell, and control both the activation and tolerance of T cells [1]. When DCs activate specific T cell responses, the specific antigen, co-stimulatory molecules (such as CD40, CD80, and CD86), inflammatory cytokines (such as interleukin-12 (IL-12), tumor necrosis factor α (TNF-α), and interferon-γ (IFN-γ)) are required for the activation of efficient cytotoxic responses [2,3]. By contrast, anti-inflammatory cytokines (such as transforming growth factor β (TGF-β), IL-10, and IL-13) and the activation of inhibitory receptors-mediated signaling pathways cause tolerogenic phenotypes of DCs [4]. Thus, the modulation of DCs is an important issue in cancer immunotherapy. Neutralizing anti-inflammatory cytokines (TGF-β, IL-10, and IL-13) via antibodies is beneficial for anti-tumor vaccination [5,6,7,8]. Cytotoxic T lymphocyte-associated molecule-4 (CTLA-4), programmed cell death receptor-1 (PD-1), and programmed cell death ligand-1 (PD-L1) are checkpoints of immune-inhibitory pathways and antibodies against CTLA-4, PD-1, and PD-L1 that have all been approved for the treatment of several types of cancers by the US Food and Drug Administration [9,10]. The other molecules involved in immune-inhibitory pathways, such as lymphocyte activation gene-3 (LAG-3, CD223), T cell immunoglobulin-3 (TIM-3), and B7 homolog 3 (B7-H3, CD276), which are considered potential targets of cancer immunotherapy, can also be targeted by antibodies [11,12,13]. With the exception of surface proteins, various intracellular proteins, including transcription factors and cytoplasmic proteins, are inversely associated with the activation of cytotoxic T immune responses; however, antibodies are not able to cross cell membranes. Alternative strategies are necessary to target these intracellular molecules in DCs.

**Figure 1:**
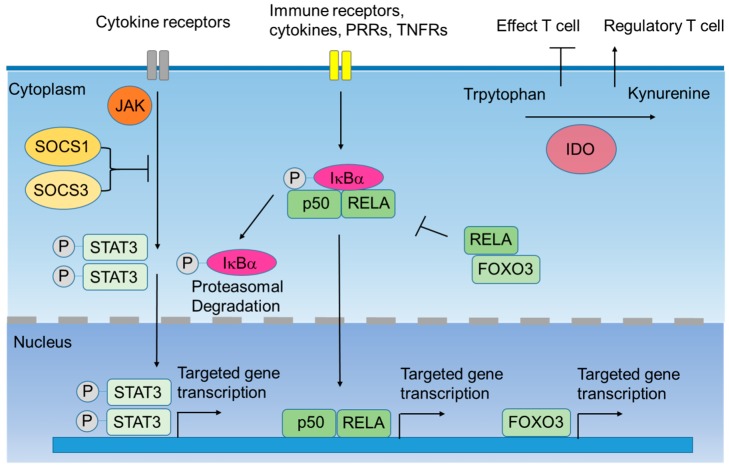
Intracellular negative immune regulators of dendritic cells (DCs). When immune receptors are triggered, downstream kinases such as Janus kinase (JAK) are activated. JAK then activates the signal transducers and activators of transcription 3 (STAT3). Nuclear translocation of phosphorylated-STAT3 (P-STAT3) activates the transcription of STAT3-targeted genes. Silenced Suppressor of Cytokine Signaling (SOCS) 1 and SOCS3 can interact with JAK and block the phosphorylation of STAT3, inhibiting the transcription of STAT3-mediated cytokines. In addition, the canonical nuclear factor-κB (NF-κB) pathway is also triggered by immune receptors. This signal leads to the phosphorylation of the IκB kinase α (IκBα), which associates with the dimers of p50 and RELA (or c-REL). Proteasomal degradation of phospho-IκBα (P-IκBα) results in the nuclear translocation of canonical NF-κB family members, which activates the transcription of downstream genes. Forkhead box O3 (FOXO3) is a transcription factor that inhibits the transcription of pro-inflammatory cytokines. Besides, cytosolic FOXO3 binds to RELA, and this complex decreases the nuclear translocation of NF-κB. Indoleamine 2,3-doixygenase 1 (IDO1) is an enzyme that degrades tryptophan into kynurenine. IDO-expressing DCs suppress the function of effector T cells and induce the expansion of regulatory T cells. Abbreviation: pattern recognition receptors (PRRS); tumor necrosis factor Receptors (TNFRs).

The utilization of short-hairpin RNA (shRNA)- and small-interfering RNA (siRNA)-based therapies is a convincing approach to silence a specific gene expression. Silencing these inhibitory molecules of DCs has been demonstrated to induce effective immune responses in several experimental models [14]. Silencing surface molecules PD-L1 and PD-L2 in DCs enhances CD8^+^ T cell proliferation and improves the efficacy of immunotherapy [15]. In addition, siRNA- and shRNA-based therapies can also target intracellular molecules. In this review, a brief outline of the cytoplasmic and nuclear targets for cancer immunotherapy is discussed (Figure 1).

## 2. Intracellular Negative Immune Regulators

### 2.1. Indoleamine 2,3-Dioxygenase-1 (IDO1)

IDO1 is a rate-limiting enzyme in the tryptophan-degrading pathway [16]. In tumor-draining lymph nodes, IDO1 expression of DCs is induced, and IDO1-expressing DCs suppress effector T cell proliferation and activate regulatory T cells because of tryptophan deprivation, downstream kynurenine, and other metabolites [17,18,19]. In addition, the ligation of CTLA-4 with the B7 family co-stimulatory molecules of CD80 and CD86 induces IDO1 expression in DCs [20]; therefore, IDO1 is an important immunotherapy target in cancer treatment. The compound 1-methyl-d,l-tryptophan (1MT) is an inhibitor of IDO1, and single-agent administration of 1MT has delayed tumor outgrowth in a transgenic model of human epidermal growth factor receptor 2 (HER2)-driven breast cancer [21]. The clinical translation of several IDO1 inhibitors, including indoximod (D-1MT) (an indirect inhibitor), navoximod (a tryptophan non-competitive inhibitor), epacadostat (a tryptophan competitive inhibitor), and BMS-986205 (an irreversible inhibitor), has been evaluated in clinical trials [22]. Preclinical data reveal that treatment of IDO pharmacological inhibitors can revert the tumor-induced immunosuppressive effect and induce anti-cancer responses [23]. Early clinical results show promising efficacy and favorable pharmacology and toxicology [24]; however, the results of a phase III study of epacadostat indicate that no survival benefit is observed with a combination of epacadostat and the anti-PD-1 antibody when compared to the PD-1 antibody alone [25]. Another phase III trial of other IDO inhibitors was stopped [25]. Thus, further research on clinical strategies and drug development will be important for IDO inhibitors. 

### 2.2. Silenced Suppressor of Cytokine Signaling (SOCS) 1 and SOCS3

Each of the proteins in the SOCS family contain a Src homology 2 (SH2) domain and a C-terminal SOCS box, which associate with ubiquitin ligase machinery [26,27,28]. The SH2 domain of SOCS directly interacts with Janus kinase (JAK) or signal transducers and activators of transcription (STAT) [29]. SOCS1 can directly inhibit JAK activity, because the SOCS1 protein contains a kinase inhibitory region (KIR) that binds with the substrate-binding groove of JAK [30]. Therefore, SOCS1 negatively regulates cytokine receptors and toll-like receptor signaling, and is involved in DCs-mediated autoimmune regulation, because SOCS1 blocks the JAK/STAT-mediated transcription of cytokine-inducible genes [31,32]. SOCS1-deficient DCs enhance the secretion of pro-inflammatory cytokines, antigen presentation capacity, and T cell proliferation when compared to SOCS1-expressing DCs [33]; thus, SOCS1 should be a negative immune regulator in DCs.

SOCS3 also contains a KIR and can directly suppress JAK activity [34,35]. Additionally, SOCS3 can inhibit IL-6 and IFN-γ signaling in DCs [36,37]. SOCS3 in DCs controls T cell activation and tolerance, because SOCS3 can bind to IDO1 and subsequently results in ubiquitin-mediated proteasomal degradation of the IDO1 protein in DCs [38]. These findings imply that SOCS3 plays a dual role in the regulation of T cell responses. 

### 2.3. IκB Kinase α (IκBα)

The transcription factor family of nuclear factor kappa B (NF-κB) consists of five subunit proteins—p65 (RELA), RELB, c-REL, p50 (NF-κB1), and p52 (NF-κB2)—which associate with both homodimer and heterodimer forms [39]. NF-κB-mediated transcription controls various physiological pathways, including inflammation through canonical and non-canonical pathways [40]. Immune receptors, such as pattern-recognition receptors, cytokine receptors, and the tumor necrosis factor receptor superfamily, activate NF-κB-mediated pathways in immune cells [41]. In addition, NF-κB is essential for the activation of plasmid DNA-induced immunity [42], while IκBα is a subunit of IκB kinase complex, and the presence of IκBα is important for the activation of the NF-κB canonical pathway [41]. The stimulation of pro-inflammatory responses results in the degradation of IκBα and the nuclear translocation and activation of NF-κB [43]; thus, silencing IκBα can be a strategy to enhance inflammatory-signaling pathways in DCs via the activation of NF-κB signaling.

### 2.4. Signal Transducers and Activators of Transcription 3 (STAT3)

IL-6 is a cytokine with a pleiotropic effect on inflammation and immune responses [44]. IL-6 stimulation induces the dimerization of interleukin 6 signal transducer (gp130) and then triggers downstream signal pathways, including the JAK-STAT3 pathway and the JAK-SHP2-mitogen-activated protein kinase (MAPK) pathway after the binding of IL-6 to IL-6R [45]. IL-6 signaling suppresses the major histocompatibility complex (MHC) class II expression through STAT3 activation in murine DCs [46]. Inhibition of JAK2/STAT3 signaling in immature DCs, via the inhibitor JSI-124, increases the expression of MHC class II and co-stimulatory molecules, the activation of antigen specific T cells, and the activation of transcription factor NF-κB signaling [47]. On the other hand, several types of toll-like receptor (TLR) agonists can activate both innate and adaptive immune responses, which can be exploited by combinations of cancer immunotherapy drugs [48]. The TLR agonist-induced immune activation can be attenuated by STAT3-mediated signaling [49]; thus, STAT3 functions as a negative immune regulator of DCs.

### 2.5. Forkhead Box O3 (FOXO3)

In mammals, the forkhead box O (FOXO) family of transcription factors (FOXO1, 3, 4, and 6) has been found [50]. FOXO3 is involved in multiple physiological processes such as metabolism, cell survival, cell death, and immune responses [51,52]. A previous study suggests that FOXO3 plays an important role in the synthetic CTLA-4–immunoglobulin-induced immune tolerance in DCs [53]. Further evidence shows that CTLA-4–immunoglobulin induces the nuclear localization of FOXO3 and then inhibits the production of pro-inflammatory cytokines [54]. Thompson et al. report that FOXO3 directly binds with NF-κB p65 (RELA) in the cytosol of DCs, and that the FOXO3–NF-κB p65 interaction decreases the transcription activity of downstream NF-κB-mediated pro-inflammatory genes [55]. Blockage of the FOXO3 signaling axis could be a target for DCs-mediated enhanced anti-tumor immunity.

## 3. Targeting Intracellular Immune Regulators Ex Vivo: DC Vaccines

Using a DC vaccine is a strategy to induce specific anti-tumor cytotoxic immune responses. DCs are isolated from the host, subsequently activated ex vivo with tumor-specific antigens, and administered as a cellular vaccine [5]. The safety of DC vaccines has been documented in many clinical trials. Early pilot studies demonstrated that ex vivo antigen-pulsed DCs can produce anti-tumor immunity against several types of solid tumors including melanoma, breast, colon, and prostate cancers [56]. However, long-term clinical efficacy of DC vaccines is still restricted to a certain population of patients; consequently, boosting DC vaccines with cytokines, adjuvants, chemotherapy, and inhibition of immune-suppressive pathways has been investigated in many studies [3]. Some of the DC vaccines targeting intracellular negative immune regulators are listed in Table 1 and will be discussed in detail below.

### 3.1. IDO1

In clinical trials, the combination of the DC vaccine sipuleucel-T and the IDO inhibitor indoximod has been evaluated. Co-administration results in a >2-fold extension in patient progression-free survival [57]. In a murine breast tumor model, DCs loaded with tumor antigens and siRNA against IDO1 decreased the tumor size and the apoptosis of CD4^+^ and CD8^+^ T cells, when compared to DCs without IDO1 silencing [58]. When human DCs are transfected with IDO siRNA, and specific tumor antigens, there is an enhanced allogeneic T cell response when compared to IDO-expressing DCs [59,60]. This suggests that IDO1-silencing enhances the efficacy of human DC vaccines.

### 3.2. SOCS1 and SOCS3

In a mice model, SOCS1 and SOCS3 expression was detected in bone marrow-derived DCs (BMDCs) [61]. When BMDCs are transfected with SOCS1 siRNA, the production of pro-inflammatory cytokines, such as TNF-α and IL-6, in DCs is enhanced. In addition, lentiviral vector (LV)-SOCS1-siRNA transduced DCs significantly enhance antigen-specific anti-tumor immunity [62]. Compared to wild type BMDCs, SOCS1-deficient BMDCs induce stronger T-helper 1 cells (Th1) responses and more effective anti-tumor immunity in vivo [63]. To enhance the therapeutic effect of a DC vaccine against cervical cancer, BMDCs infected with adenovirus-SOCS1 shRNA were pulsed with human papillomavirus (HPV) antigen E7. The results showed that SOCS1-silenced HPV E7-pulsed DCs promoted TNF-α, IL-12, and IL-6 expression; anti-E7 antibody titer; and cytotoxic activity [64]. SOCS3 might play a dual role in immune cells. In myeloid cell-specific SOCS3-conditional knockout mice, SOCS3^−/−^ macrophages simultaneously suppressed inflammation and tumor metastasis [65]; therefore, SOCS3 is still considered a therapeutic target for cancer immunotherapy in DCs [66,67]. In a phase I clinical trial, the human DCs derived from peripheral blood mononuclear cells were delivered with human SOCS1 shRNA and tumor antigens via an adenovirus vector. Using these genetically-modified DCs resulted in a complete remission rate of 83% in acute myeloid leukemia patients [68]. The therapeutic efficacy will be evaluated in subsequent clinical trials.

### 3.3. STAT3

In a murine melanoma model, BMDCs transfected with STAT3 siRNA by lipopolyplexes enhanced the maturation of DCs; the secretion of TNF-α, IFN-γ, and IL-12; and the proliferation of allogenic T cells [69,70]. In addition, the co-delivery of nanoparticle-mediated STAT3 siRNA and TLR7/8 agonist or TLR3 agonist enhanced expression of co-stimulatory CD40 and CD86 in DCs, and DCs-mediated anti-tumor immunity [71,72]. These findings suggest that STAT3 might serve as a potential target in human DC vaccines.

## 4. Targeting Intracellular Immune Regulators In Vivo: DNA Vaccines

With the exception of ex vivo targeting of DCs, the direct targeting of DCs has been researched in recent studies. Compared to ex vivo targeting, in vivo targeting is less labour-intensive and has a lower cost in large-scale production. Although in vivo targeting strategies are not specific to a single cell type of DCs or other types of cells, DCs can be activated and matured within the natural environment in hosts [73]; therefore, many studies have focused on in vivo targeting strategies, such as DNA vaccines. Tumor-specific immune responses can be induced in the tumor-bearing host by plasmid DNA encoding tumor-specific antigens or tumor-associated antigens [74]. DNA vaccines can be delivered via various routes including intramuscular, intradermal, intravenous injection; biolistic device; and oral application of attenuated bacteria [75]. Therefore, co-administration of tumor antigens and shRNA targeting in a plasmid model is an available strategy to silence immune-negative regulators in DCs in vivo. Examples of DNA vaccines targeting intracellular negative immune regulators are listed in Table 2 and discussed in detail below.

### 4.1. IDO1

When tumor-bearing mice were treated with shRNA plasmids against IDO1 via a biolistic device—a low-pressure gene gun—tumor growth was significantly delayed in several tumor models, including murine subcutaneous bladder and colon tumor models, and orthotopic and metastatic liver tumor models [76,77]. Th1 cytokines such as interferon-γ (IFN-γ) and CD8^+^ T cell-dependent, tumor-specific cytotoxic activity are induced in splenocytes in these tumor models [76,77]. The combination of IDO1 shRNA and the Her2/neu DNA vaccine has induced a better anti-tumor effect than the Her2/neu DNA vaccine alone, in an endogenous Her2/neu-overexpressing bladder tumor model [76]. Intramuscular injection of IDO1 shRNA also delayed tumor growth in a subcutaneous murine lung tumor model and increased CD11b^+^Ly6G^+^ neutrophils infiltration into the tumor [78]. Blache et al. reported that the systematic delivery of bacteria-transformed IDO shRNA significantly suppressed tumor growth and increased tumor infiltration by neutrophils in a murine melanoma tumor model [79]. It is interesting to note that, in two independent studies, the therapeutic effect of intramuscular-injected IDO shRNA and bacteria-transformed IDO shRNA was eradicated when tumor-bearing mice received a neutrophil-depleting antibody [78,79]. Neutrophils might, therefore, play a role in in vivo IDO shRNA-mediated anti-tumor responses.

### 4.2. IκBα

NF-κB activation is essential for eliciting tumor-specific cytotoxicity during intradermal DNA vaccination [42]. The delivery of plasmid DNA-encoding IκBα shRNA and a melanoma-associated antigen tyrosinase-related protein-2 (TRP2) into skin-resident DCs via intradermal injection and electroporation can further enhance TRP2-specific cytotoxic activity, delay tumor growth in a melanoma subcutaneous tumor, and reduce the number of lung melanoma foci in mice injected intravenously with melanoma [80].

### 4.3. FOXO3

The delivery of FOXO3 shRNA via a low-pressure biolistic device decreases apoptosis of DCs in vivo. In a murine bladder tumor model, the combination of FOXO3 shRNA and the Her2/neu DNA vaccine significantly enhanced CD8^+^ T cell-dependent cytotoxic activity and the anti-tumor effect in a murine tumor model [81].

### 4.4. STAT3

In a murine melanoma model, the combination of oral administration of bacteria-transformed surviving DNA vaccine and intravenous administration of STAT3 shRNA induced synergistic effect [82]. When mice were injected with STAT3 shRNA, the proliferation and granzyme B levels of infiltrating CD4^+^ and CD8^+^ T cells were induced [82].

## 5. Strategies of Improving siRNA/shRNA and Immunotherapy Efficacy

The SiRNA/shRNA silencing of intracellular negative immune regulators in DCs is a strategy to enhance anti-tumor immunity in DC vaccines and DNA vaccines. The combination of DC vaccines and immune checkpoint inhibitors, such as PD-1 and CTLA-4, is a strategy to enhance therapeutic efficacy. The co-administration of the CTLA-4 antibody and human DC vaccines co-transfected with IDO siRNA, the tumor antigen survivin, and human telomerase reversed transcriptase (hTERT) enhanced anti-tumor immunity in a patient with metastatic melanoma [83]. In addition, several ongoing clinical trials are evaluating the combination of DC vaccines (including sipuleucel-T, acute myeloid leukemia DC vaccine, and tumor lysate particle-loaded DC vaccine), DNA vaccines (DNA vaccine encoding prostatic acid phosphatase and six melanoma-associated peptides), and immune checkpoint inhibitors [84].

Novel delivery strategies might enhance anti-tumor efficacy. The ex vivo delivery of SOCS1 siRNA to BMDCs via octa-arginine (R8)-modified lipid envelope-type nanoparticles effectively delayed tumor growth [71]. Murine DCs, which were delivered with STAT3 siRNA via poly(d,l-lactic-co-glycolic acid) (PLGA) nanoparticles, led to enhanced allogenic T cell proliferation [69]. The delivery of nanovaccines containing cytosine-guanine oligodeoxynucleotides, STAT3 siRNA, and tumor-specific antigens to DCs significantly enhanced the CD8^+^ T cell responses and inhibited tumor growth [85]. These strategies might be beneficial for the development of DC vaccines in the future.

With the exception of intracellular immune-negative regulators, molecules not directly involved in immune regulation might serve as therapeutic targets. For example, silencing the pro-apoptotic molecules such as Bak and Bax via siRNA prolonged the life of DCs and enhanced the efficacy of the HPV E7 DC vaccine in murine tumor models [86]. Furthermore, the co-administration of Bak1 or Casp8 siRNA and the Her2/neu DNA vaccine significantly reduced tumor progression in a spontaneous mouse mammary tumor model [87]. Thus, identifying novel targets is still required to enhance the efficiency of DC vaccines and DNA vaccines.

## 6. Conclusions

**Figure 2:**
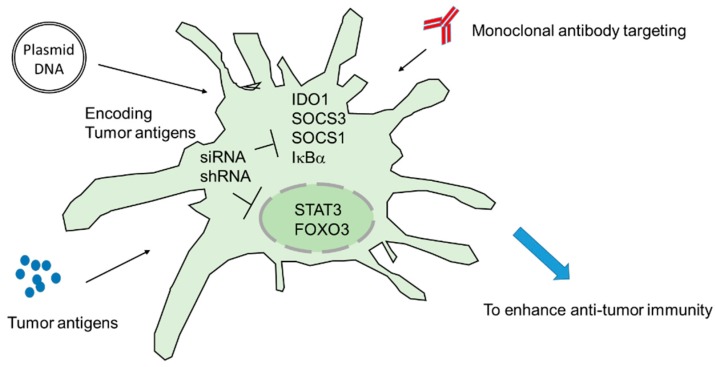
Combination of siRNA/shRNA and other cancer immunotherapies. The inhibition of the negative immune regulator in DCs results in strong immune activation. When DCs are delivered with plasmid DNA (DNA vaccine)-encoding specific tumor antigens or DCs pulsed with specific antigens, DCs-mediated anti-tumor immunity is significantly enhanced. Moreover, the monoclonal antibodies targeting CTLA-4 and PD-1 might further enhance tumor-specific immune activation.

In this article, we focused on known/potential intracellular immune regulators of DCs and introduced the application of siRNA/shRNA on DC vaccines and DNA vaccines. A summary of the combination of therapeutic strategies is outlined in Figure 2. Animal and clinical studies have revealed that the siRNA DC vaccine is safe. There are multiple ongoing clinical trials investigating the combinations of siRNA/shRNA and DC/DNA vaccines. Further investigations will lead to the development of optimal therapeutic strategies for the treatment of human cancers.

## Figures and Tables

**Table 1:** Ex vivo silencing intracellular negative immune regulators of dendritic cells.

Strategy	Species	Clinical Trial	Target Gene	Type of DC	Knockdown Method	Tumor Antigen	Tumor Type	Reference
DC vaccines	Mouse	No	IDO1	BMDC	Liposome	Tumor cell lysate	Breast cancer	58
DC vaccines	Human	No	IDO1	PBMC-derived DC	Electroporation	hTERT Survivin	Ovarian cancer	59
DC vaccines	Human	No	IDO1	PBMC-derived DC	Electroporation	No	No	60
DC vaccines	Mouse	No	SOCS1	BMDC	Lentivirus	Trp2	Melanoma	62
DC vaccines	Mouse	No	SOCS1	BMDC	Adenovirus	HPV16 E7	Ovarian cancer	64
DC vaccines	Human	Phase I	SOCS1	PBMC-derived DC	Adenovirus	Survivin Mucin1	acute myeloid leukemia	68
DC vaccines	Mouse	No	STAT3	BMDC	Nanoparticle	No	Melanoma	69
DC vaccines	Mouse	No	STAT3	BMDC	Liposome	No	Melanoma	70

DC: dendritic cell; BMDC: bone marrow-derived DC; PMBC: peripheral blood mononuclear cells; hTERT: human telomerase reverse transcriptase; Trp2: tyrosinase-related proteins 2; HPV: human papillomavirus.

**Table 2:** In vivo silencing intracellular negative immune regulators of dendritic cells.

Strategy	Species	Target Gene	Targeting Site	Knockdown Method	Tumor Antigen	Tumor Type	Reference
DNA vaccines	Mouse	IDO1	Skin	Biolistic device	Her2/neu	Bladder and colon cancer	76
DNA vaccines	Mouse	IDO1	Muscle	Intramuscular injection	No	Lung cancer	78
DNA vaccines	Mouse	IDO1	Systemic	Bacteria-transformed, intravenous injection	No	Melanoma	79
DNA vaccines	Mouse	IκBα	Skin	intradermal injection	Trp2	Melanoma	80
In vivo DNA vaccines	Mouse	FOXO3	Skin	Biolistic device	Her2/neu	Bladder cancer	81
In vivo DNA vaccines	Mouse	STAT3	Systemic	Bacteria-transformed, Oral administration	Survivin	Melanoma	82

BMDC: bone marrow-derived DC; Trp2: tyrosinase-related proteins 2; Her2: human epidermal growth factor receptor 2.
